# Proceedings of the Organon Society of Boston

**Published:** 1889-07

**Authors:** S. A. Kimball

**Affiliations:** Secretary


### PROCEEDINGS OF THE ORGANON SOCIETY OF BOSTON.

### Meeting of Organon Society, April 11th, 1889.

Dr. Wesselhœft read from the *Organon*, beginning at Section 152.

Section 152—Dr. Wesselhœft—That is, the more acute a disease, the more striking the symptoms.

Dr. Bell—Sometimes they are not so characteristic as in typhoid, when the system seems overpowered by the disease.

Dr. Defriez—We often have to go by objective symptoms alone, when the system is so overpowered as in diphtheria.

Section 153—Dr. Wesselhœft—This is one of the most instructive paragraphs in the *Organon*. Often young beginners, in reading the *Materia Medica*, will say that one remedy is exactly like the others, that all the organs of the body are affected by every remedy. But the characteristics and conditions are what must be taken into consideration. For a diagnosis of disease these peculiar and individual symptoms are not essential, but in diagnosing the remedy, an entirely different process, they are most important. For instance, two cases of acute rheumatism, in one the patient cannot bear to have the limb remain in one position, must have it changed frequently; the other patient cannot bear to have his limb moved a particle one way, has little thirst, and during the sweat must be covered up; the other may be thirsty for large quantities, in a profuse sweat, and throws the clothes off. The diagnosis is the same, but the remedy in each entirely different. In diagnosing a disease we have nothing to do with diagnosing the remedy, and the diagnosis is important in regard to the prognosis, diet, etc., but for diagnosing the remedy the name of the disease has nothing to do with it.

Dr. Bell—I would not attempt to practice Homœopathy if this were not so. Every case is a new case, and the moment we get characteristic symptoms we have something to work from, a patient that complains of sleeplessness, dizziness, presents nothing, and is a complete waste like a desert. Take vertigo, there is a list of remedies as long as your arm, but a patient, as in a recent case, that is dizzy going up-stairs and in the open air gives us the characteristics of the remedy. I sympathize with students; all remedies must seem alike to them, but any man or woman who accepts this paragraph will succeed even if they give the third potency, and they will go higher, but if they do not accept it they will only succeed by mistake. The real difficulty is that physicians do not notice these characteristic symptoms enough; the patient may not complain of them, and they do not reside in any special sphere. A child two years of age was ill with pneumonia, there was a constant rolling of the head; this led to the study of Podophyllum, which cured the case. We cannot have this paragraph enough impressed upon every one of us; it is one of the great fundamentals in the art of prescribing.

Dr. Defriez—I had a case of dyspepsia lately that had been patched up several times by so-called homœopathic treatment. A curious symptom was the sensation of a piece of ice in the pit of the stomach. He received Bovista, and the next morning had a good appetite, and there has been no return of the dyspeptic symptoms.

Dr. Wesselhœft—A great difficulty that young men have to contend with is that the patient says he don't want anything for his piles. He wants something for his stomach. Every young man who gives a remedy to such a patient without regard for this paragraph prostitutes his art every time.

Dr. Bell—Patients seem to be most disturbed by a disturbance of function; they want that cured and then they will get well afterward.

Dr. Wesselhœft—The patients are not so much to blame perhaps. They are taught this, "how can I get well until this hemorrhoid ceases to bleed, and my bowels move?"

The most harmful examples of this sort of thing, are the ideas that they must sleep; they must not suffer pain, and they must have a stool every day. These are three most tremendous lies, and have put more patients under ground than anything else. Homœopathy says you must suffer pain in order that we may get a remedy to cure it.

This is the sort of thing we have to contend with; every chronic patient needs a lecture, usually about the bowels.

What mischief the rectal diseases are doing now! Everybody is making an attack upon the piles; these are very important in the selection of a remedy, and if interfered with by operative measures will complicate the case very much. We often have to ask what the characteristics of the piles were before interference, and the suppressed symptoms will often help in selecting the remedy.

Dr. Bell—Dr. Wesselhœft's remark about the patient's wishing to be relieved from pain reminds me of a case of neuralgia that came in this evening. He wished the pain stopped to-night surely. I said it could be done; we could cut his head off.

Dr. Wesselhœft—Physicians will sometimes give a patient a remedy so he can "go somewhere to-night," but such physicians can have no love for their art.

Dr. Defriez—It seems as if homœopathic physicians did not talk enough to their patients of what Homœopathy really is.

Section 154—Dr. Wesselhœft—Hahnemann says that if we are fortunate enough to have a case with characteristic symptoms from which we have repeatedly seen results, we know that the remedy should be given, and should be let alone to do its work.

Dr. Bell—One portion of our school derides the observation of modalities, and are now trying to get a pathological materia medica, which would cut out these two paragraphs.

Dr. Wesselhœft—These two of the most important paragraphs are to be left out, and a materia medica made to suit pathological conditions.

Dr. Cobb—I had a case of colic in which I gave one dose of a well indicated remedy. The next day she was sitting up and soon recovered. She said that under Morphine treatment she was usually sick five or six weeks with attacks of the same severity.

Dr. Wesselhœft—How many of this class of cases do we have that do not recover as readily as this; what then? We must tell them that if they allow us to take them through the colic with strict homœopathic treatment they will not have the attacks as often or as severely hereafter; we can assure them of this with perfect confidence.

Dr. Bell—A recent case was that of a young lady with *dark* eyes and hair, pale, has been sick since an attack of measles two years ago. Her menses since then have been too early and profuse, and she had now been flowing seven weeks, bright-red discharge; painless, agg. on rising from a seat and walking about. Examination showed a perfectly normal condition. She had been taking hot douches with temporary relief; her appetite was poor. She craved sour things, and had been taking iron in considerable quantities. I gave Puls.$^{cm}$. The flowing stopped the next day, and the next report was that the next menstruation was perfectly normal.

Dr. Wesselhœft—This is a very interesting case, as Puls. is usually thought of with scanty menstruation, but it is one of the most important remedies for the sequelæ of measles.

S. A. KIMBALL, *Secretary.*

